# Two different pathways of phosphatidylcholine synthesis, the Kennedy Pathway and the Lands Cycle, differentially regulate cellular triacylglycerol storage

**DOI:** 10.1186/s12860-014-0043-3

**Published:** 2014-12-10

**Authors:** Christine Moessinger, Kristina Klizaite, Almut Steinhagen, Julia Philippou-Massier, Andrej Shevchenko, Michael Hoch, Christer S Ejsing, Christoph Thiele

**Affiliations:** Life and Medical Sciences Institute, University of Bonn, Carl-Troll-Str. 31, 53115 Bonn, Germany; Department of Biochemistry and Molecular Biology, University of Southern Denmark, Campusvej 55, 5230 Odense, Denmark; Max Planck Institute of Molecular Cell Biology and Genetics, Pfotenhauerstrasse 108, D-01307 Dresden, Germany

**Keywords:** Lysophosphatidylcholine, Acyl transferase, Lipid droplets, Drosophila melanogaster

## Abstract

**Background:**

Lipids are stored within cells in lipid droplets (LDs). They consist of a core of neutral lipids surrounded by a monolayer of phospholipids, predominantly phosphatidylcholine (PC). LDs are very dynamic and can rapidly change in size upon lipid uptake or release. These dynamics require a fast adaptation of LD surface. We have recently shown that two Lands cycle PC synthesizing enyzmes, LPCAT1 and LPCAT2 can localize to the LD surface.

**Results:**

Here, we show that knock-down of both enzymes leads to an increase in LD size without changes in the total amount of neutral lipids, while interference with the de-novo Kennedy pathway PC biosynthesis is associated with changes in triacylglyceride synthesis. We show that function of LPCAT1 and 2 is conserved in *Drosophila melanogaster* by the ortholog CG32699. Furthermore we demonstrate that modulation of the LD pool by LPCAT1 influences the release of lipoprotein from liver cells.

**Conclusion:**

Activity of the Kennedy pathway regulates the balance between phospholipids and neutral lipids, while the Lands cycle regulates lipid droplet size by regulating surface availability and influencing surface to volume ratio. Differences in lipid droplet size may account for differences in lipid dynamics and be relevant to understand lipid overload diseases.

**Electronic supplementary material:**

The online version of this article (doi:10.1186/s12860-014-0043-3) contains supplementary material, which is available to authorized users.

## Background

Lipids are important components of cells, with a function in cellular structure, regulation, signaling and as energy source, in particular neutral lipids. The cellular location of storage of neutral lipid is the lipid droplet (LD). LDs consist of a core of neutral lipids that is surrounded by a monolayer of phospholipids, mainly phosphatidylcholine [1-3]. Different proteins are associated with the LDs, including several enzymes of lipid metabolism [4-15].

Many metabolic disorders like diabetes and cardiovascular diseases are associated with defects in lipid metabolism and derive from additive defects in different pathways, often described as metabolic syndrome that can gradually progress into more severe diseases. The first step is usually the excess storage of lipids within different body tissues resulting in the development of obesity [16-19]. Therefore, it is crucial to understand how the storage of lipids is regulated under normal conditions.

Lipids are in a constant flux and are continuously converted into each other. Within cells they can move within membranes and between different cellular compartments. Furthermore, lipids are exchanged between different tissues. Extracellularly, the bulk of lipids is transported in lipoproteins. These lipoproteins are soluble complexes of proteins (apolipoproteins) and lipids that are transported in the circulation of vertebrates and insects and that are synthesized in the liver and intestine. They are classified into chylomicrons (CM), very low density (VLDL), low density (LDL) and high density (HDL) lipoproteins based on their apolipoprotein component and their density, which is determined by the lipid composition [20]. The major neutral lipid, triacylglycerol (TAG), is secreted from the liver and intestine in apolipoproteinB (apoB) containing lipoproteins (CM and VLDL). In contrast to other apolipoproteins, apoB is not exchangeable between lipoproteins and resides in the plasma in a lipid-associated form only. While VLDL and CM contain apoE, apoC and apoB, LDL harbors exclusively apoB. In the absence of loaded lipids apoB cannot be secreted and is rapidly degraded [21,22]. The TAG secreted as CM and VLDL mainly derives from TAG stored in cytosolic LDs [23].

Depending on cell type, nutritional status and developmental state the LD pool can vary in droplet number, size and localization within short time scales [24]. The growth of LDs and the incorporation of FAs into TAG is a fast event, typically taking just a few minutes [25,26]. Once formed, lipid droplets can fuse with each other [27] or transfer material by a slow coalescence event [28-30]. Chronic stimulation of lipolysis in adipocytes results in a fragmentation of large LDs into small LDs, which are dispersed throughout the cell [4,31-33]. These rapid changes of the LD core require a similarly rapid increase or decrease of the LD surface or an adaptation of the surface to volume ratio.

The major phospholipid of the LD surface, phosphatidylcholine, can be synthesized by three different pathways: the de-novo pathway, which is also known as Kennedy pathway, the Lands cycle and the phosphatidylethanolamine methyl transferase (PEMT) pathway, which is restricted to liver cells [34]. In the Kennedy pathway, phosphocholine is activated with cytidine triphosphate (CTP) and transferred to diacylglyceride (DAG) to form PC. These reactions are catalyzed by the cytoplasmic CTP:phosphocholine cytidylyltransferase (CT alpha) and the membrane-embedded cholinephosphotransferase or choline/ethanolamine phosphotransferase (CEPT1/CPT1) [35]. In the Lands cycle phospholipase A2 (PLA2) removes fatty acids at the *sn*-2 position of PC, which results in the formation of lysophosphatidylcholine (LPC). This can be used in a reverse reaction, the addition of a fatty acid at the sn-2 position, to yield PC. This re-acylation is catalyzed by lysophosphatidylcholine acyltransferases (LPCATs) [36]. Recently, four LPCATs were cloned and characterized [37-42]. They are all reported to localize to the ER compartment. Due to their structure they divide into two subgroups with LPCAT1 and LPCAT2 in one and LPCAT3 and LPCAT4 in the other group. LPCAT1 is reported to function in lung surfactant production, while LPCAT2 seems to be important in inflammatory reactions.

We have recently shown that diacylglycerol acyltransferase 2 (DGAT2) can localize to the surface of LDs and is active in synthesizing locally TAG stored in the core of LDs [25]. Furthermore, we have also shown that two enzymes of the Lands cycle, LPCAT1 and LPCAT2, can localize to the surface of LDs and that they can synthesize PC directly at the LD [43].

Here, we show that these proteins are important for the morphology of the cellular LD pool in different mammalian cell types and *Drosophila melanogaster*. We demonstrate that interference with both cellular PC synthesis pathways results in an increase in LD size, but due to different mechanisms. We identified the LPCAT1/2 ortholog in *Drosophila melanogaster* and demonstrate its LPCAT activity. Additionally, we show that interference with LPCAT1 influences lipoprotein particle secretion from hepatoma cells.

## Results

### Knockdown of LPCAT1 and LPCAT2 increases LD size

**Figure 1:**
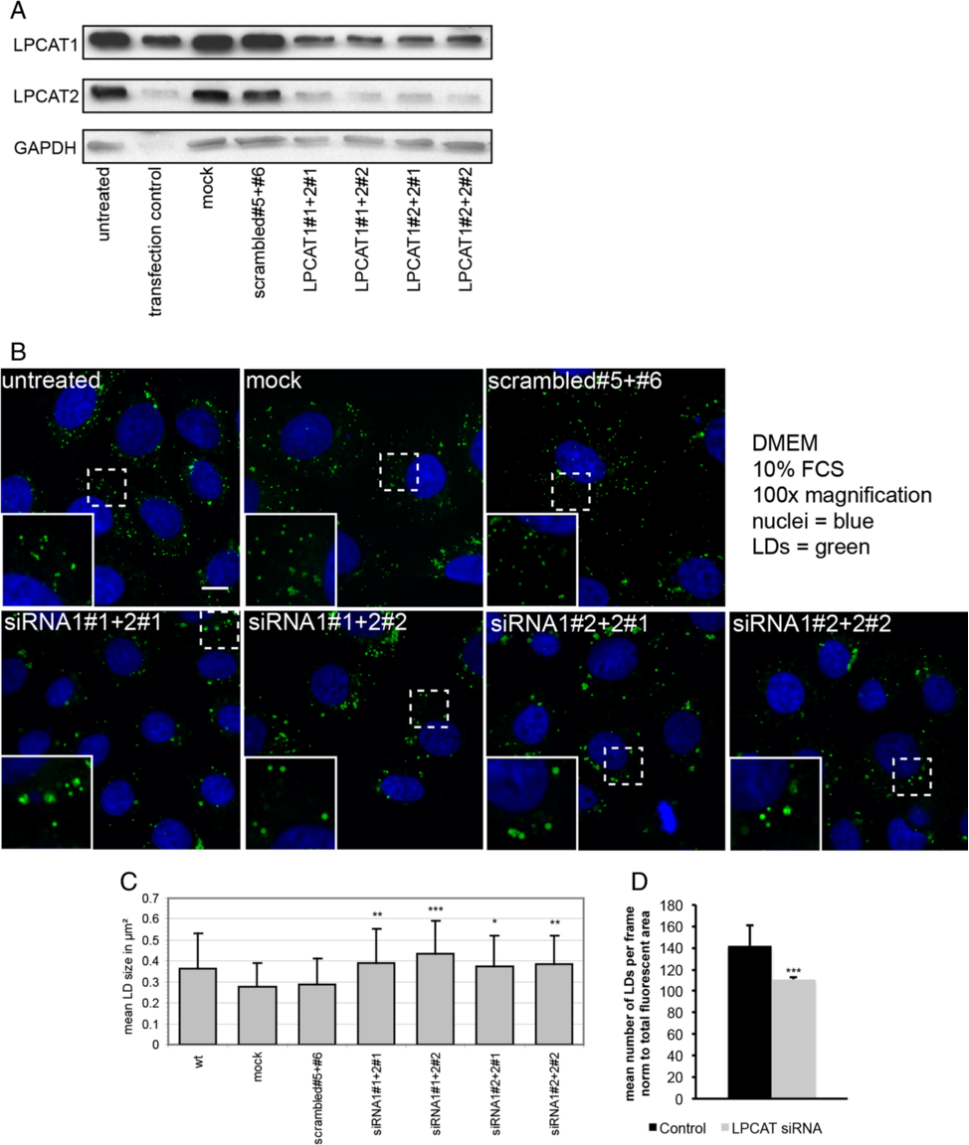
**Silencing of LPCAT1 and LPCAT2 by siRNA leads to enlarged lipid droplets in A431 cells. A)** A431 cells were either left untreated (untreated, wt), mock transfected (mock), transfected with control siRNA (eg5 as transfection control, leads to cell death, or scrambled#5 + #6 as non-targeting siRNAs) or the four possible combinations of two sequences each against LPCAT1 or LPCAT2 as indicated. After 48 h cells were lysed and subjected to SDS-PAGE/Western blotting for LPCAT1, LPCAT2 and glycerol-3-phosphate dehydrogenase (GAPDH, as a load control). **B)** Confocal images of controls and LPCAT1/LPCAT2 double-knock-downs as described in panel **A**. Nuclei (blue), LDs (green), scalebar = 10 μm. **C)** Confocal images as described in panel **B** were quantified with Image J for LD size distribution as described in Methods. Data are mean LD size ± StdDev, calculated from >50 individual cells in 3 independent experiments. Significances relative to non-targeting siRNAs were calculated by unpaired two-sided *T*-test analysis (*** p ≤ 0.001, ** p ≤ 0.01, * p ≤ 0.05). **D)** Confocal image as described in panel **B** were quantified with ImageJ. Data show mean lipid droplet number per frame, corrected for variations in cell density, calculated from >50 individual cells in 3 independent experiments. Control: scrambled#5 + #6, LPCAT siRNA: average of all siRNA treatments. Significance was calculated by unpaired two-sided *T*-test analysis (*** p ≤ 0.001).

**Figure 2:**
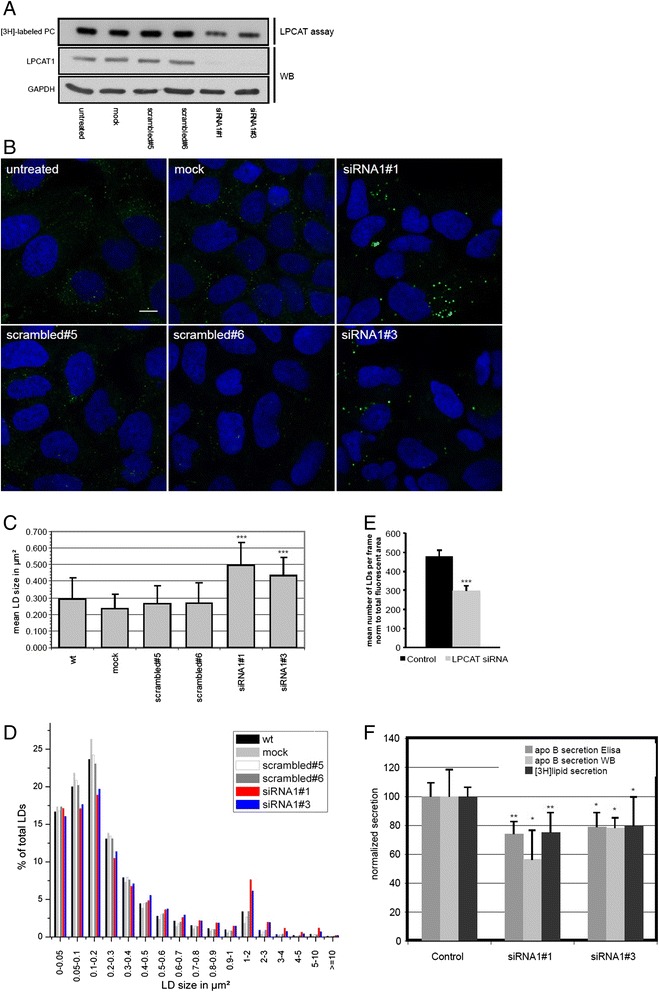
**Silencing of LPCAT1 by siRNA leads to enlarged lipid droplets and reduced lipoprotein secretion in HuH7 cells. A)** HuH7 cells were either left untreated (untreated, wt), mock transfected (mock), transfected with control siRNA (scrambled#5 or scrambled#6 as non-targeting siRNAs) or the two different siRNA sequences against LPCAT1 as indicated. After 72 h cells were subjected to a LPCAT activity assay or Western blotting (WB) for LPCAT1 using GAPDH, as load control. **B)** Confocal images of controls and LPCAT1 knock-downs as described in panel **A**. Nuclei (blue), LDs (green), scale bar = 10 μm **C + D)** Confocal images as described in panel **B** were quantified for LD size distribution as described in Methods. Data represented mean LD size ± StdDev, calculated from > 50 individual cells in 3 independent experiments. Significances were calculated by unpaired two-sided *T*-test analysis relative to non-targeting siRNA (scrambled#5) (*** p ≤ 0.001). For analysis of LD size distribution (panel **D**), LDs were grouped into size classes, and the distribution displayed as percentage of total LDs per size class. Controls (black, light and dark grey and white), siRNAs against LPCAT1 (red, blue). **E)** Confocal images as described in panel **B** were quantified with Image J. Data show mean lipid droplet number per frame, corrected for variations in cell density, calculated from >50 individual cells in 3 independent experiments. Control: average of scrambled#5 and scrambled#6, LPCAT siRNA: average of both siRNA treatments. Significance was calculated by unpaired two-sided *T*-test analysis (*** p ≤ 0.001). **F)** HuH7 cells were transfected with non-targeting siRNA (control) or different siRNA sequences targeting LPCAT1 as indicated. ApoB secretion was measured by ELISA (dark grey, data represent mean ± StdDev, n = 4) or by Western blotting (light grey, data represent mean ± StdDev, n = 5). [3H]lipid secretion after labeling with 1 μCi [3H]oleate was calculated as % of total radioactivity recovered (supernatant + cells) and normalized to control. Data represent mean ± StdDev, n = 4. p-Values were obtained by unpaired *T*-test relative to the respective control (** p ≤ 0.01, * p ≤ 0.05).

Since both LPCAT1 and LPCAT2 are present and active at the surface of LDs, we investigated their importance for the cellular LD pool. If local production of PC by LPCAT1 and LPCAT2 is functionally important for the LD monolayer, manipulation of LPCAT activity may result in a phenotypic alteration of cellular LD pools. Therefore, we performed siRNA-mediated gene silencing of the LPCATs followed by microscopic imaging of LDs, quantification of LD size and number and further characterization of the phenotype. A double knockdown (KD) of LPCAT1 and LPCAT2 by means of two different specific siRNAs targeting LPCAT1 and LPCAT2 lead to a reduction of both proteins in A431 cells (Figure 1A). This reduction resulted in a significant increase in the mean size of LDs, expressed as the cross-sectional area (Figure 1B and C) and a slightly reduced number of LDs (Figure 1D). Since double KDs are difficult and phenotypes are weak due to mutual compensation by the isoenzymes, we also analyzed the human hepatoma cell line HuH7, which expresses LPCAT3 [41] and LPCAT1, but no LPCAT2 [43]. Knockdown of LPCAT1 in HuH7 cells with two different siRNA sequences resulted in a decreased LPCAT activity in whole cell lysates (Figure 2A, upper row) and in a decrease in LPCAT1 protein down to about 10% of control (Figure 2A, middle row). Phenotypically, this KD resulted in the appearance of larger LDs (Figure 2B). A quantification of the microscopic images revealed a significant increase in the mean LD size upon LPCAT1 KD (Figure 2C). This increase is due to a shift in the LD size distribution from small LDs (50-300 nm^2^) to larger LDs (400 nm^2^- >1 μm^2^) (Figure 2D). This increase in size is paralleled by a significant decrease in number of LDs (Figure 2E). The results show that a reduction of LPCAT1 and LPCAT2 leads to a shift of the cellular LD sizes to larger LDs accompanied by simultaneous decrease in number of LDs in different cell types.

### Knock-down of LPCAT1 in HuH7 cells reduces lipoprotein particle secretion

HuH7 cells synthesize and secrete apoB containing VLDL particles [21,44] with a similar density as LDL particles (Additional file 1: Figure S1), whose assembly requires the long-chain acyl-CoA synthetase ACSL3 [45]. This protein activates fatty acids for subsequent incorporation into PC and localizes to LDs [46], suggesting that it functions together with LPCAT1 in PC synthesis at LDs. Earlier studies suggested that secretion of VLDL would depend on mobilization of TAG from LDs [23], which might be slower when small LDs are replaced by larger LDs with a smaller surface to volume ratio. Given these close connections between hepatic TAG and PC metabolism, we hypothesized that LPCAT1 activity might also influence lipoprotein assembly and secretion. To address this issue, we monitored the secretion of lipoprotein particles from HuH7 cells by measuring the amount of secreted apolipoprotein B (apoB). Knockdown of LPCAT1 with two distinct siRNAs resulted in a significant reduction of apoB secretion and a decrease in secretion of radiolabeled lipids (Figure 2F).

### LD size increase occurs without changes in the neutral lipid pool

**Figure 3:**
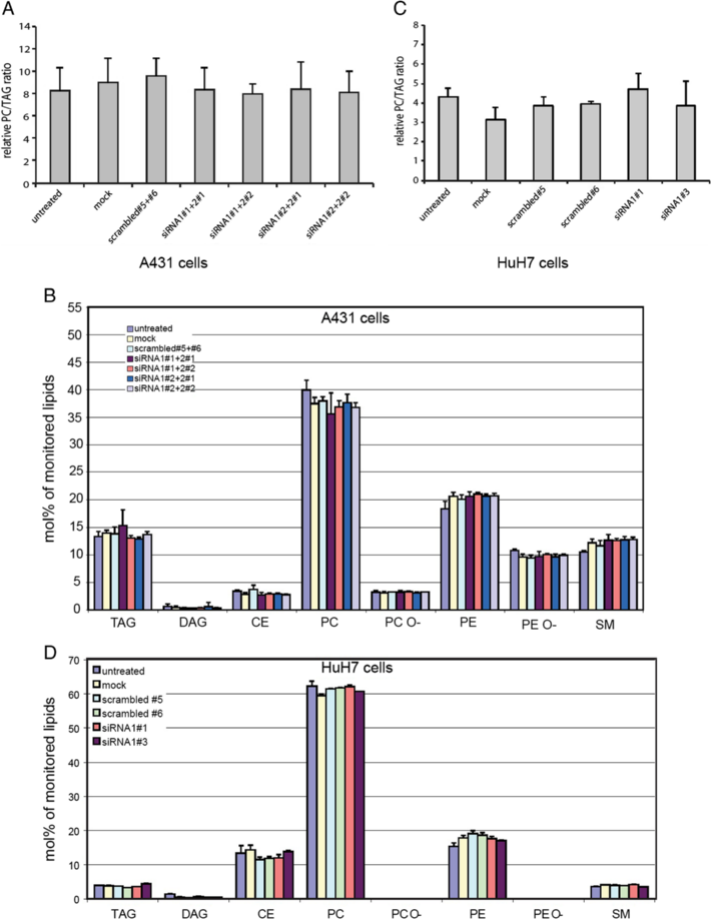
**Increase in LD size upon LPCAT1/2 knock-down is independent of neutral lipid synthesis and accumulation. A)** A431 cells were transfected with siRNA as described in Figure 1A. Seventy hours after siRNA transfection growth medium was exchanged to medium containing 10% delipidated FCS and 50 μM alkyne-oleate. After two hours cells were washed and lipids extracted. Extracts were subjected to quantitative click-analysis for the ratio of incorporation of alkyne fatty acid into TAG and PC. Data are mean ± StdDev, n = 3. Significances were calculated by unpaired two-sided *T*-test analysis relative to non-targeting siRNA (scrambled#5 + #6) and were found to be insignificant. **B)** A431 cells were transfected as described in Figure 1A. After 48 h, total lipid extracts were analyzed by mass spectrometry in duplicate samples, and species abundances were normalized to the corresponding internal standard. The molar contents of each species of the same class were summed up and normalized to the total content of all detectable lipids. **C)** HuH7 cells were transfected with siRNA as described in Figure 2A. Seventy hours after siRNA transfection as indicated, growth medium was exchanged to medium containing 10% delipidated FCS and 50 μM alkyne-oleate. After two hours, cells were washed, lipids extracted and extracts were subjected to quantitative click-analysis as in panel **A**. Data are mean ± StdDev, n = 3. Significances were calculated compared to non-targeting siRNAs (scrambled#5) and were found to be insignificant. **D)** HuH7 cells were transfected as described in Figure 2A. After 72 h, total lipid extracts were analyzed by mass spectrometry in duplicate samples. Species abundances were normalized as described for panel **B**.

An increase of LD size can result from an overall increased storage of neutral lipids, particularly TAG, or from a morphological rearrangement of the LD pool at constant TAG amounts. Neither A431 (Figure 3A) nor HuH7 (Figure 3C) cells showed a difference in incorporation of alkyne-labeled oleate into TAG relative to incorporation into PC between control siRNA-treated cells and cells treated with LPCAT1- and LPCAT2-specific siRNA. Additionally, also the amount of other major lipid species remained unchanged independent of the siRNA treatment in both cell lines, A431 (Figure 3B) and HuH7 (Figure 3D). The results suggest that the mechanism for the increase in LD size results from an adjustment of the surface to volume ratio rather than the formation of more neutral lipids.

### LD size increase by inhibition of the de-novo PC synthesis pathway is connected to changes in neutral lipid content

**Figure 4:**
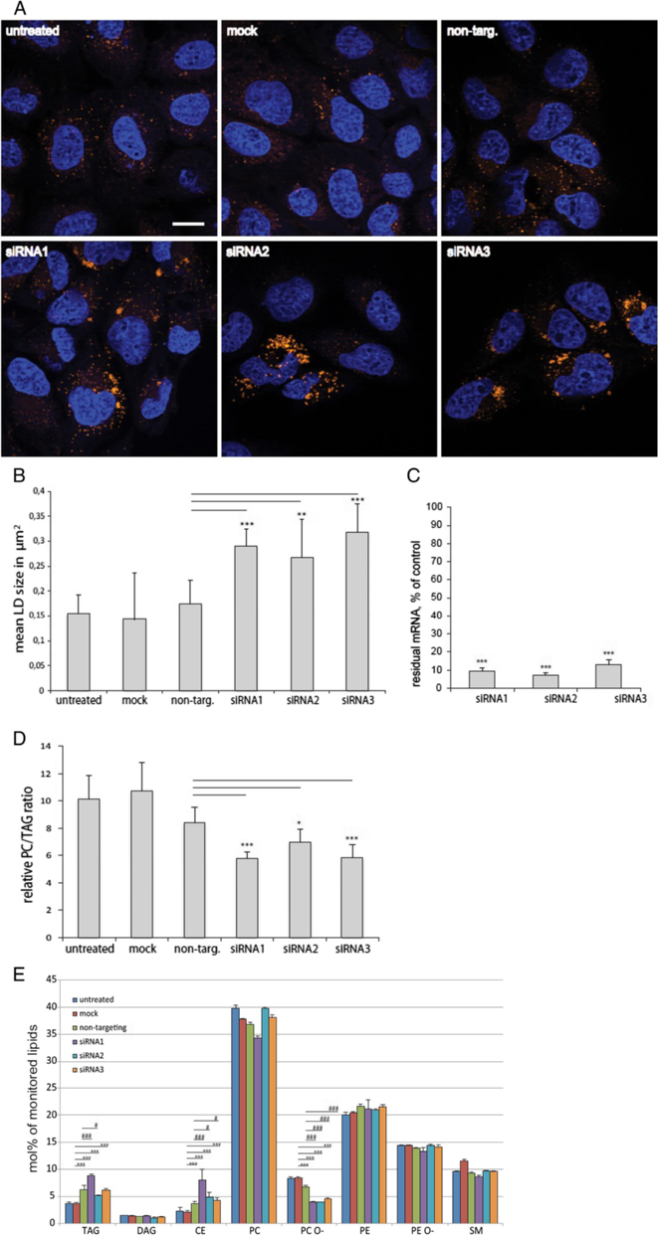
**Inhibition of de-novo PC synthesis results in increased LD size and TAG synthesis.** A431 cells were left untreated (untreated), treated with transfection reagent (mock) or transfected with a non-targeting stealth siRNAs (non-targ.) or three different stealth siRNAs against CT alpha (siRNA1-siRNA3) for 72 h. **A)** Representative confocal images, stained with DAPI (blue) and LD540 (orange). Bar, 10 μm. **B)** Images were analyzed for LD size as described in Methods. Data are mean LD size ± StdDev, calculated from > 50 individual cells in 3 independent experiments. Significances were calculated by unpaired two-sided *T*-test analysis relative to non-targeting control (non-targ.) (*** p ≤ 0.001, ** p ≤ 0.01). **C)** Amount of CT alpha mRNA left after CTalpha knock-down relative to non targeting control, measured by real-time PCR. Standard deviations and significances were calculated by unpaired *T*-test analysis from 3 different experiments (*** p ≤ 0.001). **D)** Seventy hours after siRNA transfection as indicated, growth medium was exchanged to medium containing 10% delipidated FCS and 50 μM alkyne-oleate. After two hours cells were washed and lipids extracted and analyzed for alkyne labeled TAG and PC. Data are mean ± StdDev, n = 3. Significances were calculated by unpaired two-sided *T*-test analysis relative to non-targeting control (non-targ.) (*** p ≤ 0.001, * p ≤ 0.05). **E)** A431 cells were transfected as described for panel **A**. After 72 h, total lipid extracts were analyzed by mass spectrometry in triplicate samples. Species abundances were normalized to the corresponding internal standard. Species of each class were summed up and normalized to total detectable lipids. Significances for TAG, CE and PC O- were calculated by unpaired two-sided *T*-test analysis relative to non-targeting control (### p ≤ 0.001, # p ≤ 0.05) and relative to mock transfection (*** p ≤ 0.001).

It was reported that interference with the de-novo PC pathway also results in increased LD size in S2 cells [47,48]. Furthermore, the inhibition of this pathway leads to a switch in lipid metabolism from the formation of PC towards the formation of TAG [49]. In A431 cells, we observed an increase in the size of LDs (Figure 4A,B) upon knockdown of CTalpha mRNA (Figure 4C) along with a decrease of the ratio of PC to TAG biosynthesis (Figure 4D). Steady state lipid pool size, analyzed by mass spectrometry, showed a non-significant tendency towards increased TAG and CE and a significant reduction of ether-linked PC (Figure 4E) upon CTalpha knock-down.

### LPCAT3 and LPCAT4 do not influence the cellular LD pool

**Figure 5:**
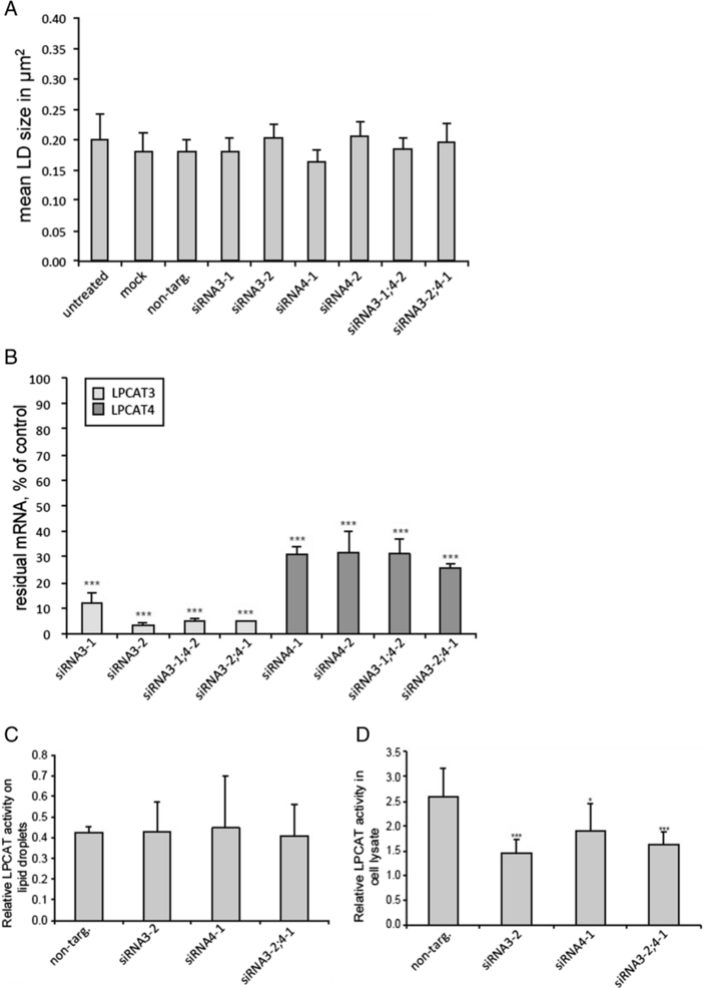
**LD size is unaffected by knock-down of LPCAT3 and LPCAT4. A)** A431 cells were either left untreated, mock transfected (mock) or transfected with a non-targeting stealth siRNAs (non-targ.) or with the two different stealth siRNA sequences against LPCAT3 (siRNA3-1, siRNA3-2) or LPCAT4 (siRNA4-1, siRNA4-2) or combinations thereof as indicated. After 72 h incubation cells were fixed, stained with DAPI and LD540 and imaged. LD size was quantified with ImageJ. Standard deviations and significances were calculated from 3 experiments by unpaired *T*-test analysis compared to non-targeting control and were found to be insignificant. **B)** mRNA was measured by real-time PCR after single or double knock-down of LPCAT3 and LPCAT4 as described for panel **A**. Values are shown relative to the non-targeting control. Standard deviations and significances were calculated as above (*** p ≤ 0.001, ** p ≤0.01). **C)** A431 cells were either transfected with non-targeting stealth siRNA or with stealth siRNA sequence against LPCAT3 (siRNA3-2) or LPCAT4 (siRNA4-1) or combination (siRNA3-2;4-1). 48 h after transfection 100 μM oleate was added to the medium to induce formation of LDs. 72 h after transfection cells were lysed and lipid droplets were purified using sucrose gradient and subjected to a LPCAT activity assay. Values are normalized to the amount of the LD protein NSDHL as determined by western blotting. Standard deviations and significances were calculated as above and were found to be insignificant. **D)** A431 cells were treated with stealth siRNA and supplemented with oleate as described for panel **C**. 72 h after transfection cells were lysed and lysate was subjected to a LPCAT activity assay using equal amounts of total protein. Standard deviations and significances were calculated from three experiments as above (*** p ≤ 0.001, * p ≤0.05) compared to non-targeting control.

Besides the homologous LPCAT1 and LPCAT2 two other proteins with LPCAT activity were identified and named LPCAT3 and LPCAT4 [41,50]. These proteins belong to a different family of proteins and are multispan transmembrane proteins structurally related to ACAT (Acyl-CoA Cholesterol Acyltransferase) proteins. Especially, the ubiquitous LPCAT3 was reported to be the main LPCAT in liver cells [41]. Though these proteins were not identified on LDs, but were reported to localize to the ER compartment, we investigated whether a single or double knock-down of these proteins has an effect on the LD pool. In A431 cells, no effect on the mean LD size (Figure 5A) was observed upon knock-down of LPCAT3 mRNA, LPCAT4 mRNA or combinations thereof (Figure 5B) Consistent with the fact that neither LPCAT3 nor LPCAT4 localize to LDs, LPCAT activities of LD preparations were unaffected by the knock-downs (Figure 5C), while total cell lysates showed significantly reduced LPCAT activities (Figure 5D). This indicates that only LPCATs with the ability to localize to LDs influence the LD size.

### The Drosophila melanogaster LPCAT1/2 ortholog CG32699 has LPCAT activity, localizes to LDs and regulates LD size

The ability to form LDs is conserved from yeast to mammals, and orthologs of many mammalian LD proteins and enzymes involved in lipid metabolism are reported in model organisms like *Drosophila melanogaster* [47,51-54]. In *Drosophila*, cutting-edge genetic technologies can be combined with immunohistochemistry, cell biology and biochemistry approaches. Furthermore, manipulations of the metabolism can be achieved by feeding specific diets that contain particular metabolites (e.g. labeled lipids) or chemical inhibitors [55-57] and the analysis of metabolite/lipid fluxes between tissues can be studied. Many functions of the mammalian liver and adipose tissue are executed in the insects fat body, an organ that is particularly pronounced in the late larval state [58].

**Figure 6:**
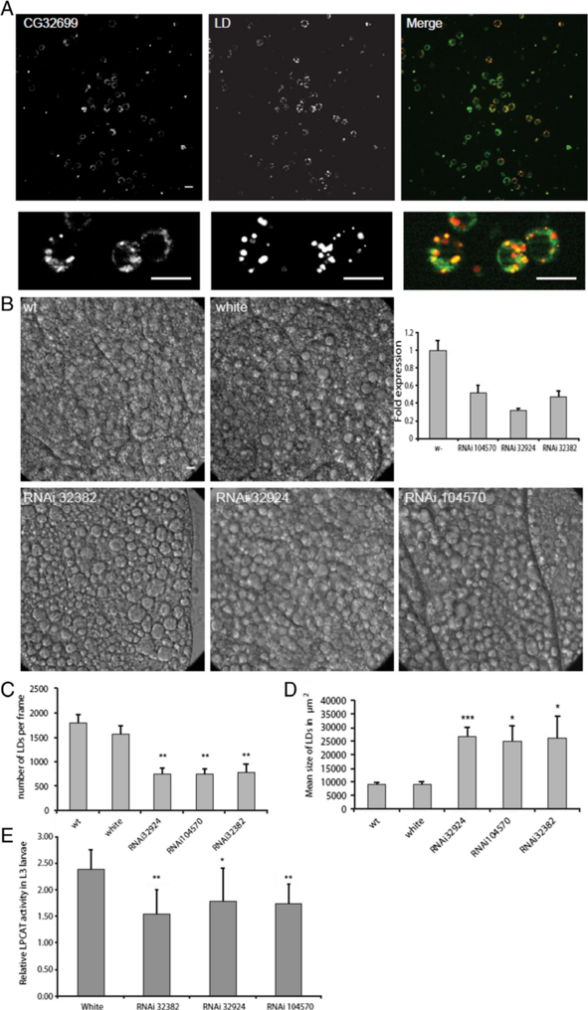
**The LPCAT1/2 fly ortholog CG32699 resembles human LPCAT1 and 2. A)** *Drosophila melanogaster* S2 Schneider cells were transfected with GFP-CG32699, which is the fly ortholog of mammalian LPCAT1 and LPCAT2. The growth medium was supplemented with 100 μM oleate. In the merged image, LDs are shown in red and CG32699 in green. Lower panels show a higher magnification of the central region of the pictures in the upper panels. Scalebar = 10 μm. Note the colocalization of GFP and LD540 signals. Resolution is limited due to the small size and round shape of S2 cells. **B)** Female virgin flies of a tubulin-Gal4 containing fly strain were crossed with male flies of three different CG32699-specific RNAi containing fly strains. L3 larvae were selected, fat bodies were isolated and representative DIC images of those fat bodies are shown for wildtype (wt), white mutant (white) and the three RNAi strains 32382, 32924 and 104570. The amount of CG32699 mRNA was measured by quantitative PCR. Scale bar = 10 μm. **C + D)** Bright field images as shown in panel **B** were quantified with ImageJ. Mean lipid droplet number per frame and mean lipid droplet size is presented for 3 frames per condition of 4 different experiments. significances were calculated by unpaired *T*-test analysis (*** p ≤ 0.001, ** p ≤0.01) compared to control. **E)** Female virgin flies of a tubulin-Gal4 containing fly strain were crossed with male flies of 3 different CG32699-specific RNAi containing fly strains or white mutant (white). From each cross three L3 larvae were selected and subjected to a LPCAT activity assay. Data were analyzed with Gel Pro Analyzer. Standard deviations and significances were calculated from three experiments by unpaired *T*-test analysis (** p ≤ 0.01, * p ≤0.05) compared to control (white).

In order to test whether the influence of the LD localizing LPCAT1 and LPCAT2 on the cellular LD pool is conserved in evolution, we investigated their function on the organismic level. LPCAT1 and LPCAT2 have one predicted ortholog in *Drosophila melanogaster*, which is annotated as CG32699 and is predicted to be a LPC acyltransferase. GFP-conjugated CG32699 in oleate-supplemented S2 cells showed colocalization with LDs (Figure 6A). Three different CG32699-specific RNAi *Drosophila* strains showed reduced levels of CG32699 mRNA (Figure 6B, inset). By DIC microscopy on isolated fat bodies of L3 larvae, we observed enlarged LDs in the fat body of RNAi strains (Figure 6B). Quantification of DIC images from RNAi strains show a reduction in the number of LDs (Figure 6C) while the size of LDs is increased (Figure 6D), similar as was seen for mammalian cells in culture. LPCAT activity assays on L3 larvae showed that LPCAT activity was decreased in the RNAi strains, indicating that this protein indeed has LPCAT activity (Figure 6E). These results show that the influence of LPCAT1/2 on the cellular LD pool is a general property of these proteins beyond cell types and species.

## Discussion

To rationalize the different effects of the above knockdowns, it is necessary to recapitulate the different isoenzymes and pathways that produce the main components of LDs, i.e. TAG and PC. In most eukaryotes, TAG is produced by DGAT1 and DGAT2. While DGAT1 is a multispan protein localizing to the ER [59], DGAT2 has a hairpin structure and localizes to both the ER and LDs [25,60,61]. A similar, but more complex situation exists for the synthesis of PC. The formation of CDP-choline by CT alpha, the rate limiting step of the PC de-novo Kennedy pathway, localizes to LDs and ER [15], but the actual synthesis of PC by CEPT1 and CPT1 is confined to the ER and Golgi [62] and not found on LDs [43]. The alternative LPCAT pathway, which forms PC from LPC and acyl-CoA (Lands cycle), has at least four isoenzymes, two of which, i.e. LPCAT3/4, are exclusively on the ER [41,63], while LPCAT1/2 again localize to ER and LDs [43,64]. Direct regulatory interactions between the pathways exist, e.g. LPCAT1 promotes the ubiquitin-mediated degradation of CPT1 [65]. Analogous to DGAT1, LPCAT3/4 are multispan proteins [41,63] while LPCAT1/2 have a hairpin structure like DGAT2 [43]. Some tissues, particularly liver, and organisms such as bakers yeast have in addition the PEMT pathway to convert PE to PC by methylation [66], which will not be discussed in more detail here.

Essentially, our data now show that reduction of LPCAT1/2 results in unchanged balance between PC and TAG synthesis, along with a remodeling of LD morphology towards larger LDs, while reduction of the de novo pathway enzyme CT alpha changes the balance between PC and TAG synthesis towards the latter, accompanied by larger LDs and higher TAG content. Reduction of ER-localized LPCAT3/4 apparently does not influence neutral lipid storage. Also, knockdown of LPCAT1 decreases lipoprotein secretion by hepatoma cells.

These observations are consistent with the following scenario: De-novo PC synthesis by the Kennedy pathway occurs mostly at the ER and depends on the availability of choline and its activation to CDP-choline by CT alpha. If other precursors and metabolic energy are available but choline is lacking, low PC synthesis capacity is balanced by increased synthesis and storage of neutral lipid [67]. The small extra amount of LD surface PC that is necessary to store the increased amounts of TAG [68] is likely recruited from other cellular membranes. Since cell growth and division requires synthesis of PC [69,70], the stored TAG can provide the precursors for PC synthesis once CDP-choline becomes available. In this situation, the rapid synthesis of PC is supported by the activation of CT alpha upon recruitment to LDs (64). The de-novo synthetic PC will be processed by the Lands cycle enzymes LPCAT1-4. Amongst those, LPCAT1/2 influence the LD size but not the amount of synthesized PC or TAG. The increase of LD size upon decrease of LPCAT1/2 activity suggests that these enzymes are either involved in the recruitment of PC to the growing LDs by vectorial acylation of the soluble precursor LPC or that their effect on PC species composition reduces the tendency of LDs to coalesce. The fact that reduction of LPCAT3/4 does not result in the same phenotype underlines the importance of compartmentalization. The different physical properties of bilayer and monolayer membranes lead to separation of these isoenzymes with polytopic and monotopic mode of membranes insertion. This allows compartmentalization of synthesis and maintenance of separate pools with different fate, but makes cells susceptible towards defects in one of the enzymes.

The complex organization of lipid metabolism is also illustrated by the role of LPCAT activity in lipoprotein secretion. Knockdown of LPCAT1 reduced lipoprotein secretion but had a very small effect on cellular lipid composition (Figure 3D). Previous findings suggested that lipidation of VLDL requires breakdown of stored TAG and transfer of released fatty acids from LDs to VLDL [23] possibly via ER-luminal LDs [71]. Therefore, it is possible that LPCAT1 influences lipid secretion by direct supply of PC for the formation of lipoprotein particles in the ER, although it appears unlikely that reduced LPCAT1 leads to a shortage of PC at the ER, where the PC de-novo synthesis pathway operates. Reduced VLDL secretion in HuH7 cells was also observed upon knock-down of ACSL3, and could be rescued by supplementation with PC [45]. Since both ACSL3 [46] and LPCAT1 localize to LDs, this supports the idea that ACSL3 supplies acyl-CoAs to LPCAT1 to generate PC on the LD surface, where about 50% of cellular LPCAT1 are found in HuH7 cells. The influence on lipid secretion may then arise from a changed lipid mobilization from LDs, where an increase in LD surface seems to be connected to increased lipolysis [31-33]. It is possible that the activity of LPCAT1 is needed to provide surface area for the LD attachment of lipid degrading and transferring proteins. By this way interference with LPCAT1 would slow down lipid secretion without interfering with the actual secretion process.

The phenotype of LPCAT1/2 depletion is identical in both, cultured cells and in the fat body of the multicellular organism *Drosophila melanogaster*. Since it is reported that many mammalian proteins and signalling pathways in lipid metabolism are conserved in *Drosophila melanogaster* (Brummer-ATGL, CG1882-CGI-58, CG11055-HSL, LSDs-PAT) [72] the similar phenotype in the mammalian cell lines and *Drosophila* fat body suggest a conserved mechanisms of LD surface control. As this single ortholog behaves similar to the mammalian proteins, it will be easier accessible for functional studies. In particular with the *Drosophila* model there is a multicellular model available, in which the importance of the protein in development or in lipid fluxes between tissues can be studied by creating a real knock-out fly.

## Conclusion

LDs are dynamic organelles that grow or shrink within minutes during periods of high FA availability or upon stimulation of lipolysis. We showed that for the growth of LDs the necessary enzymatic activities, namely DGAT2 for TAG production and LPCAT1 and LPCAT2 for surface PC production, are directly located to the surface of the LD. In line with the idea of local adjustment of LDs to growth and shrinkage, we showed that reduction of surface PC producing enzymes LPCAT1 and LPCAT2 influences lipid packaging. The restriction of LD PC synthesizing enzymes leads to increased LD size paralleled by reduced LD number, unchanged cellular neutral lipid content and reduced cellular lipid release. Our observations also emphasize the importance of isoenzymes, their different cellular localization and parallel synthesis pathways. We showed that only LPCAT1 and LPCAT2, which localize to the ER and LDs, are able to influence the LD pool while the isoenzymes LPCAT3 and LPCAT4, which localize to the ER, had no effect on the LD pool. Furthermore, we showed that interference with de-novo PC synthesis pathway also influences the LD pool leading to a similar phenotype as interference with the Land cycle, but due to a different mechanism. While interference with de-novo PC synthesis stimulates TAG synthesis and LD loading, interference of the Lands cycle PC synthesis influences lipid packing by reduction of available or appropriate surface. Our study identified an LPCAT1/2 ortholog in *Drosophila melanogaster* with similar function in regulating the cellular LD pool, emphasizing the generality and conservation of the underlying regulatory network. Our work stresses the need to understand the differences and importance of parallel synthesis pathways, isoenzymes and localization of reactions in order to understand physiological and pathological lipid storage.

## Methods

### Antibodies

Polyclonal rabbit antisera against the C-terminal peptide of human LPCAT1 CNSDAGRKPVRKKLD, conjugated to keyhole limpet hemocyanin, and against purified recombinant 6His-hLPCAT2_310-545_ were raised by Eurogentec (Seraing, Belgium) and were affinity purified against the respective antigen. Polyclonal goat anti-apoB was from Merck Bioscience, monoclonal mouse anti-GAPDH antibody was from Novus Biological, HRP-coupled polyclonal rabbit anti-goatIgG antibody from Invitrogen and HRP-coupled polyclonal goat anti-rabbitIgG and anti-mouseIgG from Dianova.

### siRNA sequences

siRNA sequences against LPCAT1-4 were from Ambion with the following order IDs:

- LPCAT1
- siRNA1#1: GGCCAGUAAGUACGGGAAAtt, ID 127470;
- siRNA1#2: CCUUCAGCUCCGUUUCAAUtt, ID 127471;
- siRNA1#3: GGACCUGCCUAAUUACCUUtt, ID 127469
- LPCAT2
- siRNA2#1: GGUAGAAGUUGAGUUUAUGtt, ID 25681;
- siRNA2#2: GCAUGAAGAGAGUACCUCAtt, ID 140447,
- against LPCAT3, stealth siRNA (Invitrogen) were used as follows
- siRNA3-1: LPCAT3 HSS115480, siRNA3-2: LPCAT3 HSS115481
- against LPCAT4, stealth siRNA (Invitrogen) were used as follows
- siRNA4-1: LPCAT4 HSS137850, siRNA4-2: LPCAT4 HSS177724
- against CT1alpha, stealth siRNA (Invitrogen) were used as follows
- siRNA1: PCYT1A HSS107689, siRNA2: PCYT1A HSS107690, siRNA3: PCYT1A HSS107691.

### Drosophila strains

Control strains Oregon-R and w^1118^ were from Bloomington Stock Center, the Gal4 driver strain tubulin-Gal4/TM3 was a gift of the Eaton Lab at MPI-CBG. UAS*-*CG32699RNAi strains (v32382, v32924 and v104570) were obtained from the Vienna *Drosophila* RNAi Center.

### Kits, reagents and chemicals

QiaShredder and RNeasy Mini Prep Kit was from Qiagen, human apoB ELISA^PRO^ was from Mabtech (Nacka Strand, Sweden), RNase-free DNase was from Roche, reverse transcriptase and RNasin were from Promega, real-time PCR Lightcycler Mix and universal probes were from Roche, primers were synthesized by Biospring, Interferin was from Peqlab, [3H]oleate was from Hartmann Analytic, the dyes 4′,6-Diamidino-2-phenylindol (DAPI) was from Sigma-Aldrich and LD540 was synthesized as described in Spandl et al. [73].

### Cell culture

A431 cells were maintained in Dulbecco’s Modified Eagle’s Medium (DMEM, Gibco 31966) supplemented with 10% fetal calf serum (FCS), HuH7 cells in RPMI (Gibco 31870) supplemented with 10 mM HEPES, 0.1 mM non-essential amino acids, 2 mM L-Glutamine and 10% FCS. Cells were grown at 37°C and 5% CO_2_.

Unless specifically indicated, media were not supplemented with additional fatty acid.

#### Drosophila Schneider 2 (S2) cells

Schneider 2 (S2) cells were cultured in Schneider medium (PAN-Biotech) supplemented with 10% FCS. Cells were grown in incubator at 28°C. *Transfection of S2 cells* The fly gene CG32699 was cloned into a UAS-EGFP (kindly provided by Bernard Fuss) containing plasmid by using Xho1 and XbaI restriction enzymes, yielding a C-terminal eGFP tagged CG32699 protein. Expression was induced by cotransfection with pMT-Gal4, which is induced by CuSO_4_. Cells were transfected in serum-free S2 medium and Cellfectin (Invitrogen) with 2 μg of each plasmid according to the manufacturers instructions. After 6 h serum-containing S2 medium was added. About 24 h after transfection the medium was replaced by serum-containing S2 medium with 500 μM CuSO_4_. Cells were processed for immunofluorescence after 24 h.

### siRNA transfection

For transfection with siRNA, cells were seeded in 24-well dishes at 5000 cells per well or 800000 per 15 cm dish and treated with Ambion siRNA (9 nM) or stealth siRNA using Interferin or Lipofectamine® 2000 (for stealth siRNA) according to the manufacturers instructions. After 48 h or 72 h cells were subjected to immunofluorescence, lipid analysis, RNA isolation or SDS-Page/Western blotting.

### Fluorescence microscopy

Cells were grown on glass coverslips and transfected with siRNA as indicated. After 48 h or 72 h, cells were fixed with 3% (w/v) of paraformaldehyde in PBS for 30 min, washed with PBS and stained with DAPI and Bodipy 493/503 or LD540 in PBS, followed by three washes in PBS. After rinsing in water, coverslips were mounted in Mowiol 4-88 containing 2.5% 1,4-diazabicyclo[2.2.2]octane. Images were acquired with a Zeiss LSM 510 confocal microscope equipped with a 100× NA 1.3 oil objective using laser excitation at 364 and 488 nm or a ZEISS Axio Observer.Z1 with a 63× NA 1.2 objective.

### Quantification of fluorescent images

Quantification was done with ImageJ particle analysis of the LD detecting channel. The threshold was set to omit the background, circularity adjusted to omit clusters and particle detection only at a size between 0,02 and 20 μm^2^. The number of particles was normalized to the total fluorescent area detected in the frame LD staining channel.

### Purification of lipid droplets and LPCAT activity assay

Cells were grown in 15 cm dishes and were supplemented with 100 μM oleate 24 h before lipid droplet isolation. Cells were washed and scraped in ice-cold disruption buffer (20 mM Hepes/NaOH, pH 7.4, 0.25 M sucrose, Roche Complete protease inhibitors) and homogenized using the EMBL cell-cracker (HGM) with 12 strokes using a maximum clearance of 18 μm. The lysate was centrifuged at 1000 g for 10 min and post-nuclear supernatant (PNS) was adjusted to 1.1 M sucrose. Two ml of adjusted PNS were overlaid with 10 ml of ice-cold disruption buffer in centrifuge tube. The gradients were centrifuged using a Beckman L-60 ultracentrifuge with the SW41 rotor at 110000 g at 4°C for 3 h. 1.5 ml of lipid droplet fraction was collected from the top. For LPCAT activity assay 10 μl of lysate (adjusted according to protein concentration measured with Bradford reagent) or 150 μl of lipid droplet fraction (adjusted according to relative amount of NSDHL measured by WB analysis) were mixed with 100 or 150 μl of assay buffer (200 mM Tris/HCl pH 7.5, 10 mM MgCl_2_, 2 mg/ml fatty acid free BSA) supplemented with 100 μM propargyl LPC (final concentration) and 20 μM oleoyl-CoA (final concentration), and subsequently incubated for 10 min at 30°C. Reaction was stopped by adding 800 μl of chloroform/methanol, 1:3, and 400 - 600 μM of 1% acetic acid in water. The phases were separated by centrifugation at 14000 × g for 30 s. The chloroform phase was collected for the click reaction, which was performed as described below.

### Lipid extraction from 24 well plate

Cells were washed twice with 1% delipidated BSA in PBS and once with only PBS. Lipids were extracted directly from the dish using first 400 μl of chloroform/methanol, 1:5 followed by 400 μl of chloroform/methanol 1/1. After addition of 600 μl of water, phases were separated by centrifugation. The chloroform phase was collected for Click reaction, which was performed as described below.

### Lipid analysis by mass spectrometry

Cell were scraped into 155 mM NH_4_HCO_3_ and were mixed with 20 μl internal lipid standard mixture providing a spike of 57 pmol PE 17:0/17:0, 47 pmol PC 18:3/18:3, 47 pmol SM 17:0, 37 pmol DAG 17:0/17:0, 37 pmol TAG 17:1/17:1/17:1 and 57 pmol CE 17:0. Samples were extracted with 990 μl chloroform/methanol (15:1) for 120 min at 4°C [74]. The apolar organic phase was isolated and evaporated. Lipid extracts were dissolved in 200 μl of 7.5 mM ammonium acetate in chloroform/methanol/2-propanol (1:2:4) and subjected to quantitative lipid analysis in positive ion mode on a QSTAR Pulsar-*i* instrument (MDS Analytical Technologies) or a LTQ Orbitrap mass spectrometer (Thermo Fisher Scientific) equipped with a TriVersa NanoMate nanoflow ion source (Advion Biosciences, Inc., Ithaca, NJ) [74-77].

### Real-time PCR

RNA was extracted into 30 μl nuclease-free water from a 24-well according to manufactures description with the RNeasy Mini Prep Kit (Qiagen). RNA (11 μl) was transcribed to cDNA using reverse Transcriptase. The cDNA was diluted 1:5 with nuclease-free water and 4 μl were used for real-time PCR. Real-time PCR was performed on a LightCycler® 480II from Roche using the universal probe system from Roche. For LPCAT3 Sonde8, for LPCAT4 Sonde48, for CTalpha Sonde 72 and as reference gene GAPDH Sonde60 were used. Sequences of the RT-PCR Primer: LPCAT3 Primer1: GGGCTACGTCTCCTTCGATT, Primer2: ATTTGTCCCACGTGAAGAGG; LPCAT4 Primer1: GCGTTGGAACCACAGCTC, Primer2: ACATAGCCAGCGGACAGC; CTalpha Primer1: GATGAGGTGGTGAGGAATGC, Primer2: TGCCAGCAGATGAATAAGGA; GAPDH Primer1: AGCCACATCGCTCAGACAC; Primer2: GCCCAATACGACCAAATCC.

Real-time PCR analysis of *Drosophila* strains was performed as described in [57]. RpL32 (rp49) Primer 1: GCTAAGCTGTCGCACAAATG, Primer2:GTTCGATCCGTAACCGATG;CG32699 Primer1: GGATGATCTCAAAGCGAAACC, Primer2: CCTTCATGGTCACATAGTGGA.

### Analysis of Drosophila melanogaster strains

Four to six L3 larvae were collected and dissected in PBS on ice. Fat bodies were transferred in PBS to object slides on ice and covered with coverslips. Microscopy was performed with a 63× NA 1.2 water objective using differential interference contrast brightfield. The images were processed and analysed with ImageJ. Processing of the image includes sharpening, despeckle and contrast enhancement. Structures were detect with the functions Find Edges, Make Binary, Morphology Tool (circle 5.00, close), Binary erode, Set Threshold, and analyse particle (circle, micron^2^, 0-infinity, exclude edges). The number and size of particles detected were selected as output data. The analysis is based on 4 independent experiments and 3 pictures per condition per experiment. The significance was calculated by unpaired students *T*-Test to wildtype fly larvae.

### Acyltransferase assay in L3 larvae using click chemistry

We applied the assay procedure as recently described [78], based on quantitative click-analysis of lipid metabolism [79]. L3 larvae were homogenized with a pestle and 60 μl of disruption buffer (20 mM HEPES/NaOH, pH 7.4, 0.25 M sucrose) was added. Then it was mixed with 40 μl of assay buffer (150 mM Tris/HCl, pH 7.5, 7.5 mM MgCl_2_, 1.5 mg/ml fatty acid-free bovine serum albumin (BSA), 250 μM oleoyl-CoA (Sigma), 250 μM propargyl LPC) and 10 min incubation at 30°C followed. Reaction was stopped by adding 500 µl of chloroform/methanol, 1:3, and 500 µl of 1% acetic acid in water. The phases were separated by centrifugation at 1900 × g for 5 min. The upper phase was removed and 70 µl of the lower chloroform phase was taken for Click reaction. Chloroform was evaporated, then 7 μl of chloroform and 30 μl of reaction mixture (2.3 mM Cu(I)BF_4_, 0.08 mM hydroxyazidocoumarin in ethanol/acetonitrile, 3.4:1) was added and 2.5 h incubation at 42°C followed. Reaction mixture was separated by TLC, first in CHCl_3_/MeOH/H_2_O/Acetic acid, 65:25:4:1 for 40 min, then in hexane/ethyl acetate, 1:1 for 30 min. The plate was dried, briefly immersed into 4% diisopropylethylamine in hexane, dried again and exposed to 420 nm light. Emission signal was detected with CCD camera and then quantified.

### ApoB secretion assays

HuH7 cells were plated in 24well plates (5000 cells per well) 16 h prior transfection. Cells were transfected with siRNAs (control siRNA scrambled#5 or #6 and siRNA against LPCAT1 siRNA1#1 and siRNA1#3) using the transfection reagent Interferin according to manufactures instructions. Transfections were performed in 100 μl OPTI-MEM and 500 μl serum-free supplemented RPMI medium. In case of radioactive quantification of lipid release, 1 μCi [3H]oleate was added to the medium per 24-well for 24 h. Then, remaining radioactivity was removed and cells were grown in 200 μl serum-free supplemented RPMI for further 48 h. In case of apoB determination by apoB ELISA, siRNAs were removed after 24 h and cells were grown for further 48 h in 200 μl serum-free RPMI. Supernatants were collected 72 h after transfection and corresponding cells were either scraped in 50 μl Lämmli buffer (apoB measurement by Western blotting) or lipids were extracted (measurement of radioactive labeled lipid release) or protein content was measured with a protein detection assay from Bio-Rad (apoB measurement by ELISA). Supernatants were either mixed with Lämmli buffer (apoB measurement by Western blotting) or lipids were extracted (measurement of radioactive labeled lipid release) or they were subjected to human apoB ELISA^PRO^ from Mabtech (Nacka Strand, Sweden). For quantification of apoB release by Western blotting, supernatant (40 μl) and cell lysate (15 μl) were subjected to SDS-PAGE and Western blotting and analyzed for apoB and LPCAT1 and GAPDH, respectively, and the amounts for apoB and LPCAT1 were normalized to GAPDH and compared to control. For characterization and quantification of released radiolabeled lipids, the extracted lipids were separated by thin layer chromatography or quantifed by scintillation counting. For quantifaction of apoB release by ELISA the amount of apoB released was normalized to the total protein content and compared to control.

## Additional file

Additional file 1: Figure S1. Density profile of apolipoprotein B containing lipoprotein particles secreted from HuH7 cells. Supernatant of HuH7 cells was separated by density gradient centrifugation based on sodium chloride and sodium bromide containing medium and the different density fractions were blotted for apoB protein.

## Abbreviations

apoB: apolipoprotein B
CM: Chylomicron
CT: CTP:phosphocholine cytidylyltransferase
CPT: Cholinephosphotransferase
DAG: Diacylglyceride
FA: Fatty acid
HDL: High-density lipoprotein particle
KD: Knock-down
LD: Lipid droplet
LDL: Low-density lipoprotein particle
LPC: Lysophophatidylcholine
LPCAT: Lysophosphatidylcholine acyltransferase
PC: Phosphatidylcholine
TAG: Triacylglyceride
VLDL: Very low-density lipoprotein particle
