# Supplementary material for: Two different pathways of phosphatidylcholine synthesis, the Kennedy Pathway and the Lands Cycle, differentially regulate cellular triacylglycerol storage
Source: BMC Cell Biol. 2014 Dec 10;15:43. doi: 10.1186/s12860-014-0043-3 (PMC4293825; doi:10.1186/s12860-014-0043-3)
Supplement: Additional file 1: Figure S1. — Density profile of apolipoprotein B containing lipoprotein particles secreted from HuH7 cells. Supernatant of HuH7 cells was separated by density gradient centrifugation based on sodium chloride and sodium bromide containing medium and the different density fractions were blotted for apoB protein. [file 12860_2014_43_MOESM1_ESM.pdf]

Additional File 1

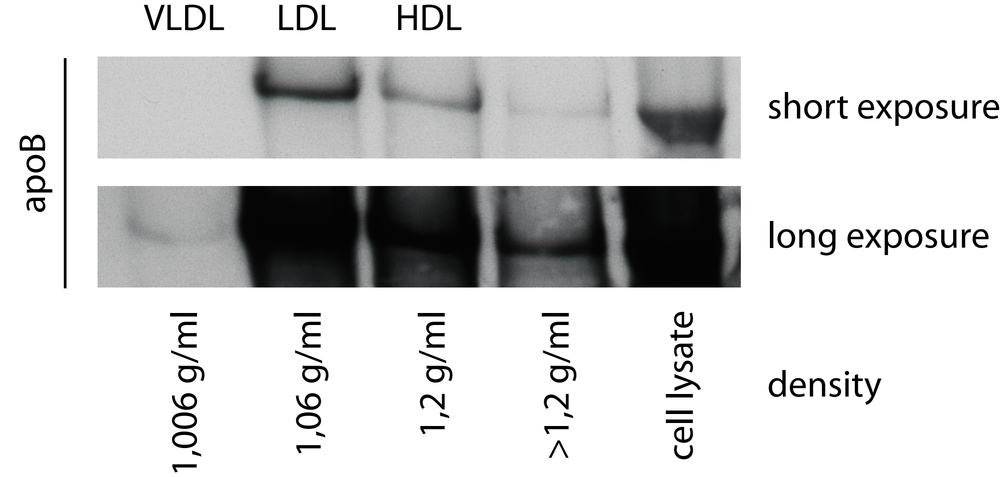

Density profile of apolipoprotein B containing lipoprotein particles secreted from HuH7 cells
Supernatant of HuH7 cells was speparated by density gradient centrifugation based on Sodiumchloride and sodium bromide containing medium and the different density fractions were blotted for apoB protein.
